# Thermodynamic and computational analyses reveal the functional roles of the galloyl group of tea catechins in molecular recognition

**DOI:** 10.1371/journal.pone.0204856

**Published:** 2018-10-11

**Authors:** Tomoya Takahashi, Satoru Nagatoishi, Daisuke Kuroda, Kouhei Tsumoto

**Affiliations:** 1 Department of Bioengineering, School of Engineering, The University of Tokyo, Hongo, Bunkyo-ku, Tokyo, Japan; 2 Global R&D, Health Care Food, Kao Corporation, Bunka, Sumida-ku, Tokyo, Japan; 3 Institute of Medical Science, The University of Tokyo, Shirokanedai, Minato-ku, Tokyo, Japan; 4 Medical Device Development and Regulation Research Center, School of Engineering, The University of Tokyo, Hongo, Bunkyo-ku, Tokyo, Japan; University of Hyderabad School of Life Sciences, INDIA

## Abstract

Catechins, biologically active polyphenols in green tea, exhibit various biological activities, such as anticancer and antiviral activities, arising from interactions with functional proteins. However, the molecular details of these interactions remain unclear. In this study, we investigated the interactions between human serum albumin (HSA) and various catechins, including some with a galloyl group, by means of isothermal titration calorimetry (ITC), differential scanning calorimetry (DSC), and docking simulations. Our results indicate that the galloyl group was important for recognition by HSA and was responsible for enthalpic gains derived from a larger buried surface area and more van der Waals contacts. Thus, our thermodynamic and computational analyses suggest that the galloyl group plays important functional roles in the specific binding of catechins to proteins, implying that the biological activities of these compounds may be due in part to the physicochemical characteristics of the galloyl group.

## Introduction

Catechins are the major functional components of green tea (*Camellia sinensis*), and some of these polyphenolic compounds show biological activities, including anticancer and antiviral activities [1, 2], resulting from their interactions with proteins [3–9]. A detailed understanding of molecular properties of catechins could lead to applications of catechins in medicine and food chemistry. Although there have been many experimental studies focusing on catechins [5, 8–10], the molecular details of the interactions between tea catechins and the proteins involved in their biological activities have not been fully investigated.

**Fig 1:**
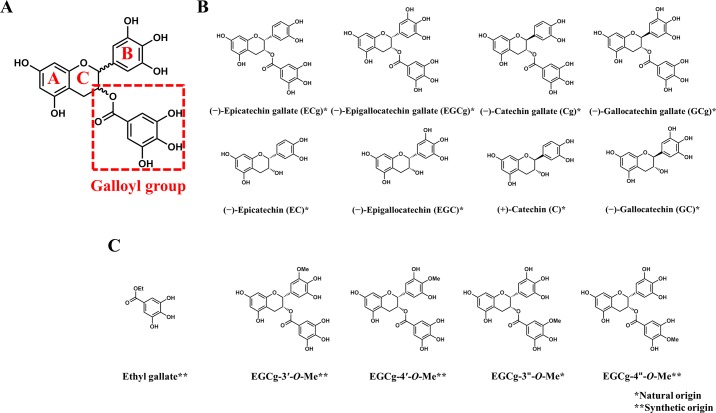
Chemical structures of catechins. (A) Chemical structure of the functional groups in catechins, (B) natural epicatechins (top row) and synthetic catechins (bottom row), and (C) ethyl gallate and analogs of the galloyl group and catechinsepigallocatechin gallate.

The chemical structure of catechins comprises A, B, and C rings, and some catechins bear a galloyl group (Fig 1A). The main catechins isolated as natural products include (−)-epicatechin (EC), (−)-epigallocatechin (EGC), (−)-epicatechin gallate (ECg), and (−)-epigallocatechin gallate (EGCg); in addition, various non-natural catechins have been synthesized, including (+)-catechin (C), (−)-gallocatechin (GC), (−)-catechin gallate (Cg), and (−)-gallocatechin gallate (GCg) (Fig 1B). Both the natural and the non-natural catechins have hydroxyl groups (two or three) at the 3′-, 4′-, and/or 5′- positions of the B ring and a hydroxyl group at the 3-position of the C ring. Epimerization at the 2-position of the C ring can be induced by heat or changes in pH [11]. Investigation of the interactions between proteins and various catechin derivatives and the stereoisomers can be expected to lead to a better understanding of the mechanisms by which these compounds are recognized by proteins.

Human serum albumin (HSA), which is the most abundant protein in plasma, interacts with various ligands [12, 13]. For example, flavonoids, including catechins derivatives, interact with HSA [14,15] and are transported to various tissues, resulting in various biological activities. Although it has been already reported that catechins interact with HSA [15,16] and the interaction could increase the cross-reactivity of EGCg with natural IgM antibodies in mouse serum [17], the molecular-level details of how catechins are recognized by HSA remain unclear. Understanding structure–activity relationships requires an understanding of the thermodynamics of ligand–protein interactions, and the combination of isothermal titration calorimetry (ITC) and spectroscopic techniques has been used to investigate the interaction between HSA and (+)-catechin [18]. The thermodynamic parameters obtained from ITC suggested that hydrogen bonds and van der Waals forces are the major binding forces in the interaction. However, only a single catechin was studied, and the molecular details of the interaction were not addressed. Various other experimental techniques, such as mass analysis with a quartz-crystal microbalance (QCM), fluorescence quenching measurements, high-performance affinity chromatography, and affinity capillary electrophoresis, have shown that the galloyl group of catechins interacts strongly with HSA [19–21]. However, most of these previous studies of the protein–catechin interactions were phenomenological and lacked atomic-level insights into the interactions.

In this study, we used HSA and 12 catechins (Fig 1) as a model system to investigate the molecular mechanisms by which proteins recognize catechins. Specifically, we performed thermodynamic analyses of the HSA–catechin interactions, as well as the docking simulations. Thermodynamic analysis is actively used in pharmaceutical research [22, 23], but it is less widely employed in studies about interactions between HSA and catechins. Our strategy involving the combination of thermodynamic analysis with a computational method provided information about the molecular basis of the HSA–catechin interactions, highlighting the importance of the galloyl group in the activity of catechins.

## Materials and methods

### Chemicals

EGCg, ECg, GCg, Cg, EGC, EC, and GC were purchased from Kurita Water Industries Ltd. (Tokyo, Japan). (+)-Catechin, Na_2_HPO_4_·12H_2_O NaH_2_PO_4_·2H_2_O, NaCl, and ibuprofen were purchased from Wako Pure Chemicals Co. (Tokyo, Japan). Ethyl gallate (EtGa) was purchased from Tokyo Chemical Industry Co. (Tokyo, Japan). (−)-Epigallocatechin-3′-*O*-methylether gallate (EGCg-3′-*O*-Me), (−)-epigallocatechin-4′-*O*-methylether gallate (EGCg-4′-*O*-Me), (−)-epigallocatechin-3′′-*O*-methylether gallate (EGCg-3′′-*O*-Me), and (−)-epigallocatechin-4′′-*O*-methylether gallate (EGCg-4′′-*O*-Me) were purchased from Nagara Science Co. Ltd. (Gifu, Japan). Warfarin was purchased from Nacalai Tesque (Kyoto, Japan). HSA was purchased from Sigma-Aldrich Japan (Tokyo, Japan). We used a non-degreased HSA. The HSA was dissolved in a phosphate buffer and the HSA stock solution was prepared by extensive overnight dialysis at 4°C. The purity of HSA was checked by using SDS-PAGE.

### Isothermal titration calorimetry (ITC)

The binding of the catechins and its analogs to HSA was examined in a VP-ITC instrument (Microcal Northampton, MA). In a typical experiment, the calorimetry cell was filled with a solution of HSA (50 μM) in a buffer composed of 50 mM sodium phosphate (pH 6.8). Catechins or its analogs were titrated to a solution of HSA at 298 K at 180-s intervals with stirring at 307 rpm. The data were analyzed with the Origin software (ver. 7) by means of a single-binding-site model. The thermodynamic parameters were obtained from three replicate titrations, where the dilution heats of the ligands were subtracted. Changes in heat capacity (Δ*C*_p_) were estimated from the slopes of linear least-squares plots of Δ*H* versus temperature in the range from 288 to 303 K measured in 5 K increments.

To obtain information about the HSA binding site of catechins, we carried out competition assays using ITC. Warfarin was used as a marker for monitoring site I of HSA. The calorimetry cell was charged with the HSA–EGCg complex ([HSA] = 50 μM, [EGCg] = 500 μM) or the HSA–EGC complex ([HSA] = 50 μM, [EGC] = 500 μM) in a pH 6.8 buffer composed of 50 mM sodium phosphate and 1% DMSO. Warfarin solution (500 μM in the same buffer) was loaded into a syringe. Warfarin was titrated into the cell containing either HSA or an HSA-EGCg complex at 180-s intervals with constant stirring at 307 rpm. The total amount of heat obtained for each measurement was taken as the total heat quantity (Δ*Q*), and we verified whether warfarin and the catechins bound to the same site by comparing the Δ*Q* from the interaction between HSA and warfarin. The binding inhibition rate was defined by the equation belowcalculated as follows:

$$\mathrm{Binding}\;\mathrm{inhibition}\;\mathrm{rate}\;(\%)=100-\frac{\Delta Q\;(\mathrm{HSA}-\mathrm{ligand}\;\mathrm{complex})}{\Delta Q\;(\mathrm{HSA}\;\mathrm{only})}\times 100$$

### Differential scanning calorimetry (DSC)

The thermal stabilities of HSA and the HSA–ligand complexes were determined by differential scanning calorimetry in a VP-DSC autosampler instrument (GE Healthcare). The cell was heated from 303 to 363 K at a rate of 1 K min^−1^. The concentrations of HSA and the ligands were 50 and 500 μM, respectively, in a buffer composed of 50 mM sodium phosphate (pH 6.8). The data analysis was carried out with the Origin software (ver. 7) using a two-state model.

### Docking simulations

Using RDKit software [24], we generated fifteen conformers for each catechin. Based on the 50 conformers for each catechin and HSA (PDB ID: 1AO6) as the target protein, we performed molecular docking simulations with RosettaScripts and the talaris2014 scoring function [25]. Starting structures were prepared by manually placing each catechin at site I or site II of HSA, respectively. For each docking simulation, 2000 putative HSA–catechin complex structures were generated, and the structures were ranked by interface score. Shape complementarity as indicated by the *S*c measure, was calculated using the Rosetta libraries [26] as described previously [27]. Accessible surface area was computed with the NACCESS program [28].

## Results and discussion

### Structure–activity relationships for HSA–catechin interactions

To characterize the HSA–catechin interactions on the basis of the catechin structures and thermodynamic data, we used ITC to analyze the interactions between HSA and the eight catechins shown in Fig 1B. To focus on the roles of the galloyl group, we also analyzed the interaction between HSA and EtGa, which was a derivative of the galloyl group (Fig 1C). The ITC profiles showed that the number of binding sites (*N*) for the eight catechins and EtGa ranged from 1.0 to 1.2 (Table 1), suggesting that all of them interacted specifically to a binding site on HSA. Our ITC results also showed that the catechins having the galloyl group (EGCg, ECg, GCg, and Cg) had higher binding affinities than the catechins without a galloyl group (EGC, EC, GC, and C) (S1A Fig, Table 1). The binding affinity of EtGa was lower than the affinities of the catechins having the galloyl group (S1B Fig), suggesting that it was only in the context of the entire catechin molecule that the galloyl group improved HSA binding affinity. The difference in binding affinities between catechins with and without a galloyl group were similar to the results previously reported [15, 21], although the ITC profiles of catechins without a galloyl group were not high reliability due to their low affinities. Therefore we discussed the thermodynamics for the interactions of eight catechins. The thermodynamic profiles revealed that the binding of EGCg and ECg was enthalpy-driven, whereas the binding of the other catechins was entropy-driven. It is tempting to speculate that the difference between the enthalpy-driven binding (such as that for ECg and EGCg) and the entropy-driven binding (such as that for EC and EGC) was due to the differences between the chemical structures of the catechins, that is, to the presence of the galloyl group. Although the binding of Cg and GCg, which are epimers of ECg and EGCg, respectively, was also entropy-driven, the Δ*H* values for the former two compounds were nevertheless more favorable than the values for EC and EGC, which lack the galloyl group (Table 1). Furthermore, the Δ*H* of the interaction between EtGa and HSA was more favorable than the Δ*H* values for the interactions between HSA and the catechins lacking the galloyl group. Our ITC analyses suggest that the presence of the galloyl group resulted in a more favorable Δ*H*, and the stereochemistry of the catechins, especially that of the galloyl group, was an important factor determining the thermodynamics of the HSA–catechin interactions.

**Table 1:** Parameters for binding of catechins and ethyl gallate to human serum albumin.

Compound	*N*	*K*_D_ (μM)	Δ*H* (kcal mol^−1^)	−*T*ΔS (kcal mol^−1^)	Δ*G* (kcal mol^−1^)	Δ*H*/Δ*G* (%)
EGCg^a^	1.1 ± 0.1	2.2 ± 0.3	−4.7 ± 0.6	−3.0 ± 0.6	−7.7 ± 0.1	61
ECg ^a^	1.0 ± 0.1	1.1 ± 0.1	−5.1 ± 0.5	−3.1 ± 0.5	−8.1 ± 0.1	63
GCg^a^	1.2 ± 0.2	8.8 ± 3.4	−3.0 ± 0.1	−3.9 ± 0.3	−6.9 ± 0.2	43
Cg ^a^	1.0 ± 0.1	3.5 ± 0.5	−2.8 ± 0.6	−4.6 ± 0.7	−7.4 ± 0.1	38
EGC ^a^	1.0 ± 0.2	20 ± 16	−0.6 ± 0.4	−5.8 ± 0.7	−6.3 ± 0.4	10
EC ^a^	1.0^b^	49 ± 18	−0.8 ± 0.2	−5.1 ± 0.4	−5.8 ± 0.2	13
GC ^a^	1.0 ^b^	52 ± 16	−1.1 ± 0.5	−4.7 ± 0.7	−5.8 ± 0.2	19
C ^a^	1.0 ± 0.2	5.0 ± 8.6	−0.3 ± 0.1	−6.8 ± 0.7	−7.1 ± 0.6	4
EtGa ^a^	1.0 ^b^	46 ± 23	−1.4 ± 0.1	−4.5 ± 0.4	−5.9 ± 0.3	24

^a^ Each value is the average of at least three independent measurements.

^b^ The binding stoichiometry was fixed to *N* = 1.

To further characterize the catechin binding to HSA, we exploited DSC to investigate the thermal stabilities of HSA in the presence and absence of the catechins. Thermal stabilities of ligand-bound HSAs were expected to be higher than those of unbound HSA [29]. Previous studies have shown that catechins could control the biological activities of certain proteins by physically binding to them and thereby improving their thermal stability [30, 31]. In line with these observations, our DSC results clearly showed that the catechins enhanced the thermal stability of HSA, as indicated by its melting temperature (*T*_m_), and that there was a moderate correlation between thermal stability and binding affinity (*R* = 0.52) (S2 Fig, S1 Table). In addition, the catechins having the galloyl group showed higher *T*_m_ values than those without a galloyl group. These results suggest that the catechins with the galloyl group bound to HSA more tightly, which resulted in a more stable HSA–catechin interaction. Thus, the galloyl group may control the biological activity of proteins through its physical interactions with them.

### Driving forces for the HSA–catechin interactions

To elucidate the main driving forces for the HSA–catechin interactions, we conducted ITC analyses in the presence of NaCl: if binding affinity is increased by the addition of NaCl, hydrophobic interactions can be considered as the main driving force for binding, whereas if binding affinity is decreased by the presence of NaCl, the main driving force is electrostatic interactions [32]. To elucidate the specific role of the galloyl group, we focused on the following six compounds: EGCg, ECg, GCg, EGC, EC, and EtGa.

With addition of 0.2 M NaCl, the binding affinities of EGCg, ECg, and GCg decreased, whereas the affinities of EGC, EC, and EtGa increased (S3 Fig, S2 Table). Therefore, the driving force for binding of the catechins having the galloyl group (EGCg, ECg, and GCg) was mainly electrostatic interactions, whereas that for EGC, EC, and EtGa was mainly hydrophobic interactions. These results imply that when present in a catechin molecule, the galloyl group enhances the electrostatic interactions between the catechin and the protein.

### Roles of the galloyl group in molecular recognition

Our ITC data clearly showed that the catechins having the galloyl group bound to HSA more strongly than did the catechins without a galloyl group (Table 1). To investigate the role of the galloyl group in more detail, we performed interaction analyses for EGCg derivatives with a methoxy group either in the galloyl moiety or in the B ring (Fig 1C). The ITC profiles showed that the number of binding sites (*N*) of these methylated catechins ranged from 0.9 to 1.1, suggesting that these compounds interacted specifically to a binding site on HSA (S4 Fig, Table 2). Although the enthalpic gains for all the methylated compounds were larger than the enthalpic gain for EGCg, no improvement in binding affinity was observed for derivatives with a methyl group in the B ring (EGCg-3′-*O*-Me and EGCg-4′-*O*-Me) owing to entropic penalties (Table 2). In contrast, despite showing similar entropic penalties, binding affinity improvements due to larger enthalpic gains were observed for derivatives with a methoxy group in the galloyl group (EGCg-3′′-*O*-Me and EGCg-4′′-*O*-Me); this result implied that the galloyl group of catechins has more potential in improving binding affinities than the B-ring in molecular recognition. The interaction between EGCg-3′′-*O*-Me and HSA showed the most increased affinity (*K*_D_ = 0.20 μM, compared to 2.2 μM for HSA–EGCg). To seek an explanation for this result, we experimentally calculated Δ*C*_p_ values, which have been shown to correlate with changes in the size of the protein–ligand interface: specifically, a more negative Δ*C*_p_ means a larger change in the interface size [33, 34]. Plots of Δ*H* versus temperature showed that the Δ*C*_p_ values for EGCg and EGCg-3′′-*O*-Me were −148 and −195 cal mol^−1^ K^−1^, respectively, suggesting that the interface of the HSA–EGCg-3′′-*O*-Me complex was larger than that of the HSA–EGCg complex (S5 Fig, S3 Table). The only difference between EGCg and EGCg-3′′-*O*-Me is the methyl group at the 3′′-position of the galloyl group (Fig 1C). Therefore, the higher binding affinities of the catechins having the galloyl group were due to the ability of the galloyl group to increase the size of the HSA–catechin interface.

**Table 2:** Parameters for binding of methylated catechin derivatives to human serum albumin.

Compound	*N*	*K*_D_ (μM)	Δ*H* (kcal mol^−1^)	−*T*ΔS (kcal mol^−1^)	Δ*G* (kcal mol^−1^)
EGCg	1.1 ± 0.1	2.2 ± 0.3	−4.7. ± 0.6	−3.0 ± 0.6	−7.7 ± 0.1
EGCg-3′-*O*-Me	0.9 ± 0.1	1.6 ± 0.2	−9.3 ± 0.4	1.4 ± 0.3	−7.9 ± 0.1
EGCg-4′-*O*-Me	0.9 ± 0.1	1.7 ± 0.1	−7.0 ± 0.1	−0.8 ± 0.1	−7.9 ± 0.1
EGCg-3′′-*O*-Me	1.0 ± 0.1	0.14 ± 0.02	−7.9 ± 0.9	1.4 ± 0.9	−9.4 ± 0.1
EGCg-4′′-*O*-Me	1.0 ± 0.1	1.0 ± 0.1	−9.8 ± 0.1	1.6 ± 0.1	−8.2 ± 0.1

### Catechin binding site on HSA

Although we showed that the catechins physically bound to a site on HSA, the nature of the binding site remains unclear. HSA has two pockets, site I and site II, where numerous small compounds have been shown to bind [12, 13]. Previous competition assays and molecular modeling studies suggest that EGCg binds to site I on bovine serum albumin, whose structure is highly homologous with that of HSA [35, 36]. Warfarin also binds to HSA at site I, as indicated by X-ray crystallography [37]. To determine the binding site of catechins on HSA, we used ITC to perform competition assays with warfarin, in which the total heat (Δ*Q*) derived from the HSA–warfarin interaction was compared with Δ*Q* values for interactions between HSA–EGCg or HSA–EGC complex and warfarin, and a binding inhibition rate was calculated as described in Materials and Methods. These assays showed that Δ*Q* values for the interactions between the HSA–catechin complexes and warfarin were smaller than the Δ*Q* for the HSA–warfarin interaction alone, resulting in smaller binding inhibition rates (S6 Fig). These observations suggest that the binding sites of warfarin, EGCg, and EGC were identical.

### Origin of the enthalpic contribution of the galloyl group

**Fig 2:**
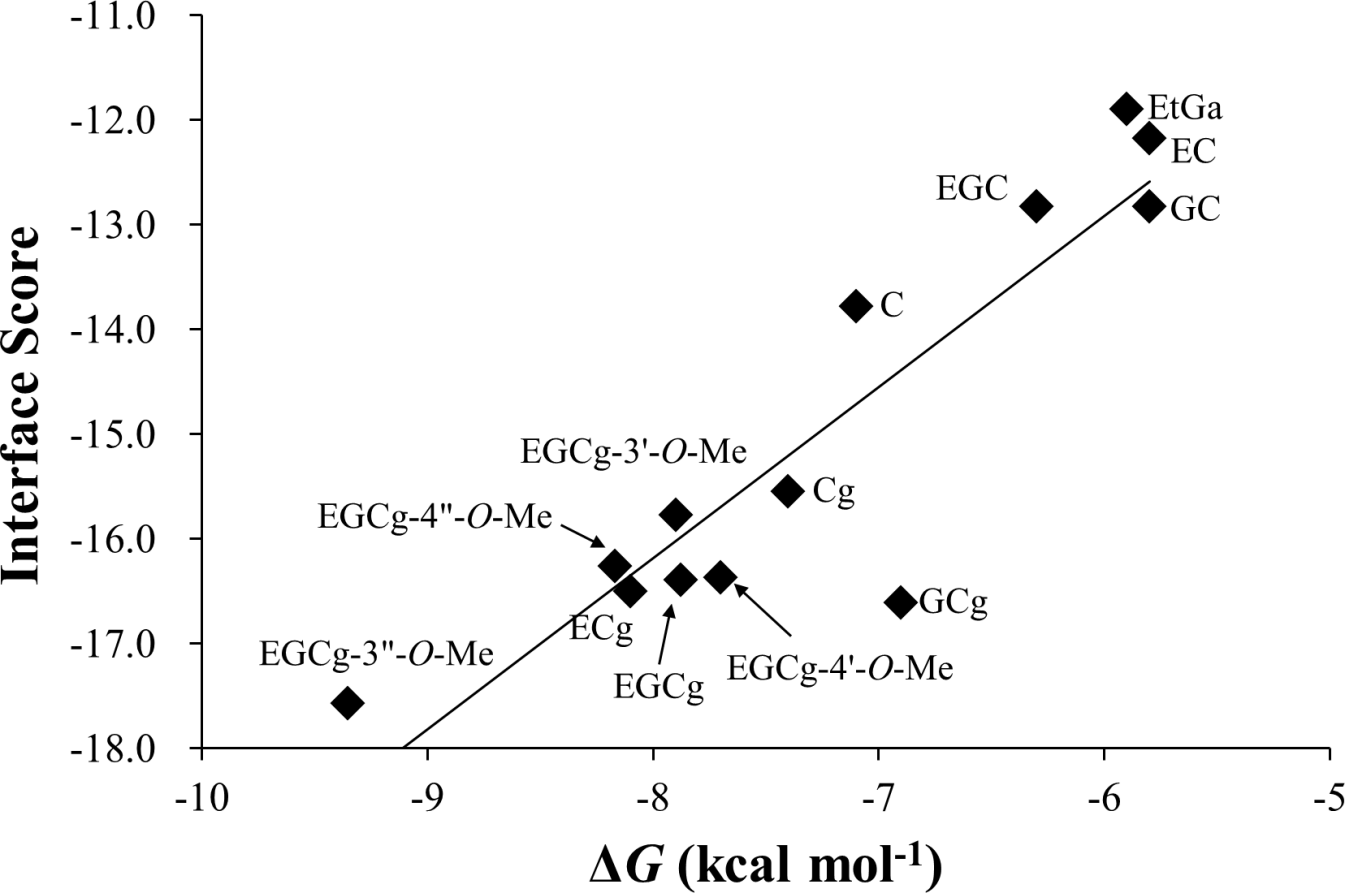
Correlation between Δ*G in vitro* and interface score *in silico* for binding of catechins and ethyl gallate to human serum albumin.

The thermodynamic analyses described above suggested that the galloyl group of the catechins contributed mainly to the enthalpic gains in the molecular recognition and that the catechins interacted specifically with site I of HSA. One of the best methods for further characterizing the molecular details of protein–ligand interactions is X-ray crystallography. However, such method is often laborious and time-consuming. An alternative is docking simulations that model protein–ligand complexes based on unbound-state structures. Hence, to further interpret the thermodynamic profiles at the molecular level, we employed docking simulations [25] between HSA site I and the 12 catechins, as well as EtGa. For each catechin, we plotted the relationship between the interface scores of the docking simulations and the experimental Δ*G* values from the thermodynamic analysis (Fig 2). A bottleneck in docking simulations occurs in the orientation and conformational sampling of a ligand in the binding site of a protein. To handle this bottleneck, we averaged the interface score over the models with the 10 top scores (S4 Table). We found that the correlation between interface score and experimental Δ*G* was very high (*R* = 0.90); this high correlation implies that, to a reasonable extent, the scoring function used in the docking simulations were able to capture the physicochemical principles behind the HSA–catechin interactions (Fig 2).

**Fig 3:**
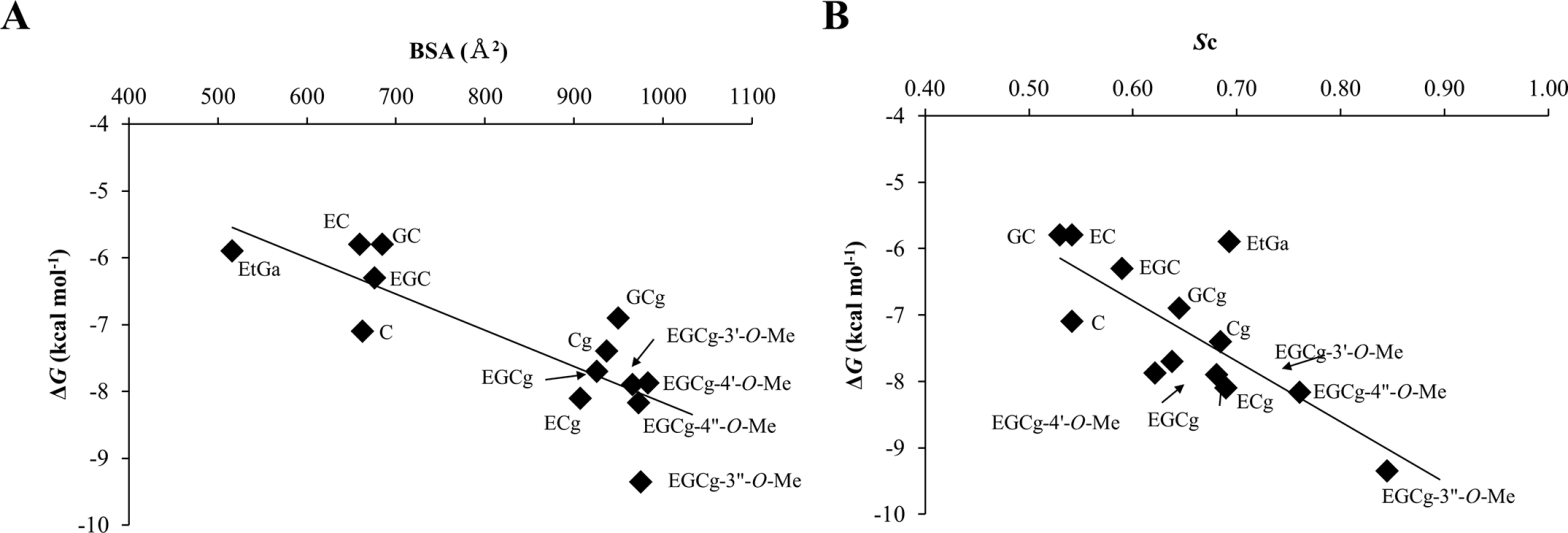
**Correlations between buried surface area (BSA), shape complementarity (*S*c), and Δ*G* for catechins and ethyl gallate:** (A) plot of Δ*G* versus BSA and (B) plot of Δ*G* versus *S*c.

**Fig 4:**
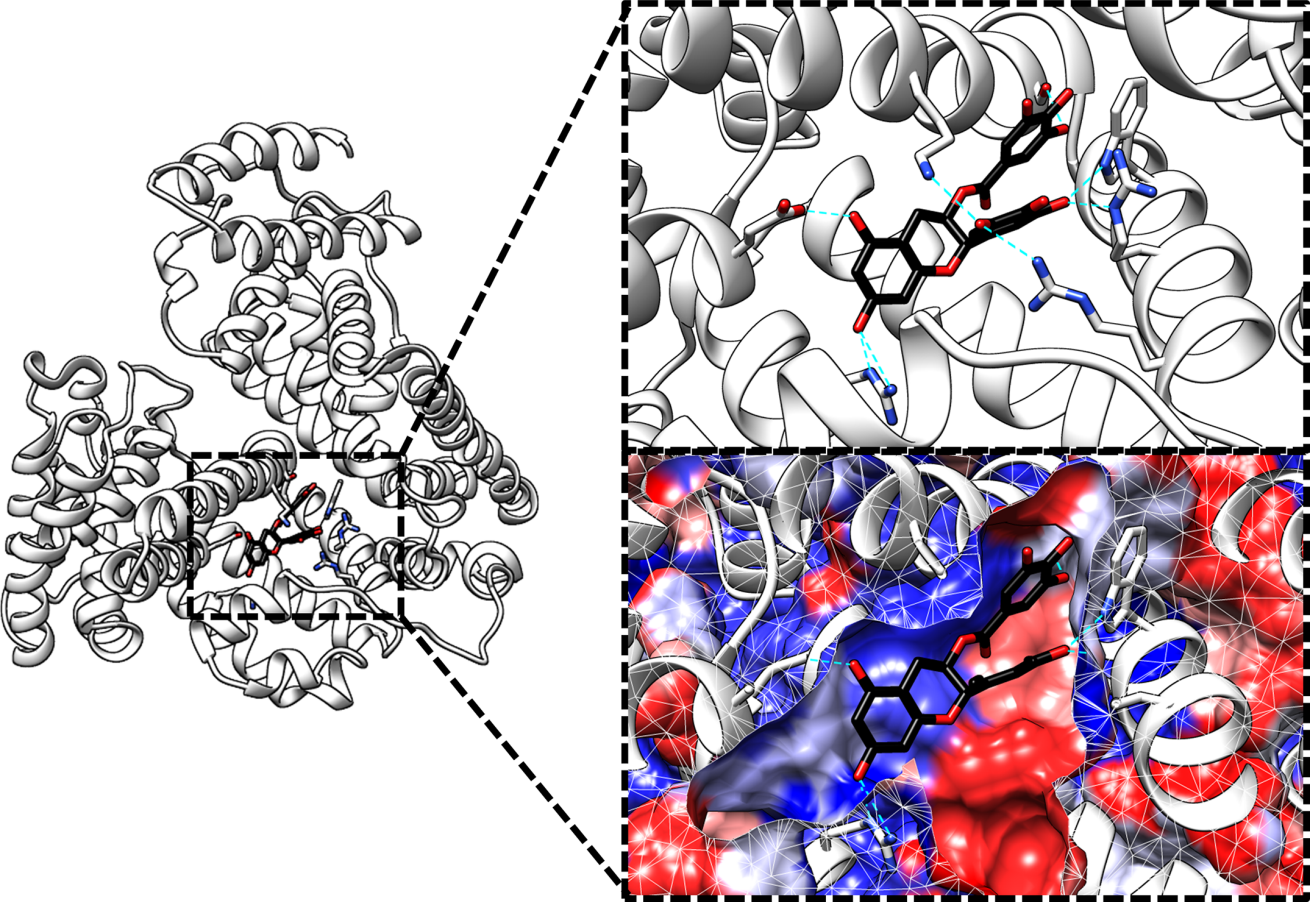
Model of a complex between human serum albumin (white) and (−)-epigallocatechin gallate (black) obtained by means of a docking simulation. Only residues that formed hydrogen bonds with EGCg were shown. Hydrogen bonds were described as cyan dotted lines. The molecular surface of the protein was colored based on the Kyte–Doolittle hydrophobicity scale (blue for hydrophilic areas and red for hydrophobic areas [38]). The figure was made by the UCSF Chimera [46].

The structures of the model complexes in which the catechins bound to site I of HSA, as well as subsequent buried surface area (BSA) calculations, showed that catechins having the galloyl group had larger BSAs than the catechins lacking the galloyl group; the correlation between BSA and experimental Δ*G* was high (*R* = 0.82, Fig 3A). Furthermore, EGCg and the methylated derivatives of the galloyl group displayed the larger shape complementarity (*S*c) than the other catechin derivatives; and shape complementarity was also well correlated with the experimental Δ*G* (*R* = 0.76) (Fig 3B). The model of the HSA–EGCg complex obtained from the docking simulations is shown in Fig 4. Hydrophobicity mapping using the Kyte–Doolittle scale [38] suggests that site I of HSA has both apolar and polar regions, so that the galloyl group of EGCg could form a hydrogen bond as well as well-packed van der Waals contacts with the molecular surface of site I (Fig 4). Thus, these results indicate that the galloyl group of the catechins increased their binding affinity by increasing the BSA and the shape complementarity, primarily by means of well-packed van der Waals interactions with the apolar/polar surface of site I of HSA.

The docking scoring function used in this study consists of several physics-based and empirical terms [25]. To better interpret the docking results, we decomposed the docking scores into their individual score terms. Because our experimental analyses relied heavily on physicochemical principles, we focused on two physics-based terms, that is, the electrostatic contribution (if_X_fa_elec) and van der Waals contributions (if_X_fa_atr and if_X_fa_rep). We found that catechins having the galloyl group had more-negative values than catechins without a galloyl group, both for the electrostatic and van der Waals interactions (S7 Fig). This result suggests that the catechins having the galloyl group tended to exploit these interaction forces more than the other catechins did, and this result is in agreement with our ITC data on the HSA–catechin interactions in the presence and absence of NaCl. Furthermore, the binding affinities measured by ITC were highly correlated with the hydrogen bond energies calculated in Rosetta [25] (S8 Fig, *R* = 0.94), which suggests that hydrogen bonds were key determinants of the interactions between HSA and catechins.

Finally, to confirm that the catechins bind specifically to site I of HSA and to determine how well site II could accommodate the catechins, we also performed docking simulations with site II. The interface scores of the docking simulations for site I were better than those for site II, implying that the catechins bound more favorably bind to site I, which is consistent with the result of our competition assays showing that the catechins bound to site I (S9 Fig).

### Importance of galloyl groups in food chemistry and drug discovery

In this study, we investigated the thermodynamic profiles of the interactions between HSA and 12 catechins, and our results indicate the importance of the galloyl group in the molecular recognition of these compounds. This information can be expected to be useful in investigations of the interactions between catechins and proteins other than HSA. Taste is one of the important factors determining the palatability of food. There are five basic taste sensations: sweetness, saltiness, sourness, bitterness and umami. Compounds with galloyl groups are reported to have a strong bitter taste [39]. Bitter compounds activate human bitter taste receptors (hTAS2Rs) [39, 40], but although several proteins related to the bitterness have been identified, how such compounds bind to the protein receptors at the molecular level is not clear. To control bitterness and thus improve the palatability of food, an understanding of the molecular details of the interactions is essential, and our results would help to unveil the physicochemical principles behind the protein–catechin interactions.

Compounds having galloyl groups have already been used as medicines; for example, tannic acid (S10 Fig), which has multiple galloyl groups, has been used to control diarrhea [41]. In the intestinal tract, tannic acid forms an insoluble coating and binds to proteins, inhibiting inflammation, which results in an astringent effect [42]. Moreover, some small molecules with galloyl groups, including a potential anticancer agent, bind to human neutrophil elastase and inhibit its function [43]. To develop drugs that bind specifically to target proteins involved in human diseases, one must optimize the binding affinities and specificities to the target proteins based on structure–activity relationships [44]. Although HSA is a reservoir protein for numerous compounds and we should carefully verify the extrapolating our HSA-catechin interaction to the potential drug-target protein interaction, our strategy based on physicochemical principles and computational methods may become a powerful tool for designing small-molecule inhibitors and drugs with galloyl groups. In addition, the molecular-level details regarding the function of the galloyl group elucidated in this study may provide us insight leading to target validation for biological functions and to the discovery of natural and synthetic compounds bearing galloyl groups with useful pharmaceutical activities.

## Conclusion

In summary, our thermodynamic analyses and docking simulations suggest that the galloyl group in tea catechins improves the binding affinity of their binding to HSA; the improvements are the result of larger enthalpic gains for the catechin having the galloyl group, gains derived mainly from larger BSAs, better shape complementarity, and higher hydrogen-bonding propensity. This work is the first to report the physicochemical significance of the galloyl group and the molecular basis of its involvement in protein recognition. Although various reports have shown that the galloyl group of catechins interacts with proteins [10, 45], the molecular details of the binding mechanism were unclear. Our results revealed the functional roles of the galloyl group in molecular recognition at an atomic-level resolution and may facilitate the discovery of new proteins involved in the biological activities of catechins; specifically, screening proteins by means of thermodynamic analyses that focused on Δ*H* generated by the galloyl group would be a plausible approach.

In this study, we focused on the interactions between HSA and 12 catechins as a model system. To generalize our findings and further explore the roles of the galloyl group of catechins, the next logical step would be to extend our analyses to proteins other than HSA, and further work in this area is underway in our laboratory.

## Supporting information

S1 Fig Comparison of the heats of HSA-ligands and the dilution heats of ligands. (A) EGCg-HSA, (B) EGCg-buffer, (C) ECg-HSA, (D) Ecg-buffer (E) GCg-HSA, (F) GCg-buffer (G) Cg-HSA, (H) Cg-buffer, (I) EGC-HSA, (J) EGC-buffer, (K) EC-HSA, (L) EC-buffer, (M) GC-HSA, (N) GC-buffer, (O) C-HSA, (P) C-buffer, (Q) EtGa-HSA, (R) EtGa-buffer.
(TIF)

S2 Fig Thermal stability of HSA upon ligand binding. (A) DSC curves for HSA unfolding in the presence of ligands.
(B) Distribution of the binding affinity and thermal stability.
(TIF)

S3 Fig Binding of Catechins and EtGa to HSA in the presence of 0.2M NaCl. (A) EGCg, (B) ECg, (C) GCg, (D) EGC, (E) EC, (F) EtGa
(TIF)

S4 Fig Binding of EGCg and methylated EGCg derivatives to HSA: (A) EGCg, (B)EGCg-3-*O*-Me, (C) EGCg-4′-*O*-Me, (D) EGCg-3"-*O*-Me, and (E) EGCg-4"-*O*-Me Click here for additional data file. (TIF)

S5 Fig Plot of Δ*H* versus temperature for EGCg and EGCg-3"-*O*-Me. (TIF)

S6 Fig Binding inhibition rates between HSA–EGCg or HSA–EGC complexes and warfarin. (TIF)

S7 Fig Correlation between electrostatic interactions and van der Waals in Rosetta. (TIF)

S8 Fig Correlation between the effect of affinity in the presence of NaCl and hydrogen bond energy in Rosetta. (TIF)

S9 Fig Correlations between Δ*G* and interface score in silico for (A) site I and (B) site II of HSA. (C) Competitive binding experiments between warfarin (binds at site I) and ibuprofen (binds at site II) and EGCg Click here for additional data file. (TIF)

S10 Fig Chemical structure of tannic acid. (TIF)

S1 Table Thermal stabilities and binding affinities of HSA and HSA–catechin complexes. (PDF)

S2 Table Binding affinities of catechins and EtGa for HSA in the presence and absence of NaCl. (PDF)

S3 Table Temperature dependence of binding parameters for the interaction of EGCg and EGCg-3”-O-Me with HAS. (PDF)

S4 Table Δ*G* and interface scores for the interaction of catechins and EtGa with HSA. (PDF)

## References

[1] Khan N, Mukhtar H. Tea polyphenols for health promotion. Life Sci. 2007;81(7):519–533. 10.1016/j.lfs.2007.06.011 17655876 PMC3220617

[2] Mori S, Miyake S, Kobe T, Nakaya T, Fuller SD, Kato N, et al Enhanced anti-influenza A virus activity of (-)-epigallocatechin-3-O-gallate fatty acid monoester derivatives: effect of alkyl chain length. Bioorg Med Chem Lett. 2008;18(14):4249–4252. 10.1016/j.bmcl.2008.02.020 18547804

[3] Kowalinski E, Zubieta C, Wolkerstorfer A, Szolar OH, Ruigrok RW, Cusack S. Structural analysis of specific metal chelating inhibitor binding to the endonuclease domain of influenza pH1N1 (2009) polymerase. PLoS Pathog. 2012;8(8):e1002831 10.1371/journal.ppat.1002831 22876177 PMC3410856

[4] Tanaka T, Ishii T, Mizuno D, Mori T, Yamaji R, Nakamura Y, et al (-)-Epigallocatechin-3-gallate suppresses growth of AZ521 human gastric cancer cells by targeting the DEAD-box RNA helicase p68. Free Radic Biol Med. 2011;50(10):1324–1335. 10.1016/j.freeradbiomed.2011.01.024 21277973

[5] Bohin MC, Roland WSU, Gruppen H, Gouka RJ, van der Hijden HTWM, Dekker P, et al Evaluation of the bitter-masking potential of food proteins for EGCG by a cell-based human bitter taste receptor assay and binding studies. Journal of agricultural and food chemistry. 2013;61(42):10010–10017. 10.1021/jf4030823 24093533

[6] Bulic B, Pickhardt M, Mandelkow E. Progress and developments in tau aggregation inhibitors for Alzheimer disease. J Med Chem. 2013;56(11):4135–4155. 10.1021/jm3017317 23484434

[7] de Freitas V, Mateus N. Protein/polyphenol interactions: past and present contributions. Mechanisms of astringency perception. Current Organic Chemistry. 2012;16(6):724–746. 10.2174/138527212799958002

[8] Ozdal T, Capanoglu E, Altay F. A review on protein–phenolic interactions and associated changes. Food Res Int. 2013;51(2):954–970. 10.1016/j.foodres.2013.02.009

[9] Tachibana H, Koga K, Fujimura Y, Yamada K. A receptor for green tea polyphenol EGCG. Nature structural and molecular biology. 2004;11(4):380 10.1038/nsmb743 15024383

[10] Braicu C, Ladomery MR, Chedea VS, Irimie A, Berindan-Neagoe I. The relationship between the structure and biological actions of green tea catechins. Food Chem. 2013;141(3):3282–9. 10.1016/j.foodchem.2013.05.122 23871088

[11] Ishino N, Yanase E, Nakatsuka S. Epimerization of tea catechins under weakly acidic and alkaline conditions. Biosci Biotechnol Biochem. 2010;74(4):875–877. 10.1271/bbb.90884 20378961

[12] Ghuman J, Zunszain PA, Petitpas I, Bhattacharya AA, Otagiri M, Curry S. Structural basis of the drug-binding specificity of human serum albumin. J Mol Biol. 2005;353(1):38–52. 10.1016/j.jmb.2005.07.075 16169013

[13] Wang ZM, Ho JX, Ruble JR, Rose J, Ruker F, Ellenburg M, et al Structural studies of several clinically important oncology drugs in complex with human serum albumin. Biochim Biophys Acta. 2013;1830(12): 5356–5374. 10.1016/j.bbagen.2013.06.032 23838380

[14] Pal S, Saha C. A review on structure-affinity relationship of dietary flavonoids with serum albumins. J Biomol Struct Dyn. 2014;32(7):1132–1147. 10.1080/07391102.2013.811700 23815082

[15] Trnková L, Boušová I, Staňková V, Dršata J. Study on the interaction of catechins with human serum albumin using spectroscopic and electrophoretic techniques. Journal of Molecular Structure. 2011;985(2):243–250. 10.1016/j.molstruc.2010.11.001

[16] Maiti TK, Ghosh KS, Dasgupta S. Interaction of (-)-epigallocatechin-3-gallate with human serum albumin: fluorescence, fourier transform infrared, circular dichroism, and docking studies. Proteins. 2006;64(2):355–362. 10.1002/prot.20995 16705651

[17] Hatasa Y, Chikazawa M, Furuhashi M, Nakashima F, Shibata T, Kondo T, et al Oxidative Deamination of Serum Albumins by (-)-Epigallocatechin-3-O-Gallate: A Potential Mechanism for the Formation of Innate Antigens by Antioxidants. PLoS One. 2016;11(4):e0153002 10.1371/journal.pone.0153002 27046229 PMC4821561

[18] Li X, Wang S. Study on the interaction of (+)-catechin with human serum albumin using isothermal titration calorimetry and spectroscopic techniques. New Journal of Chemistry. 2015;39(1):386–395. 10.1039/C4NJ01344A

[19] Ishii T, Ichikawa T, Minoda K, Kusaka K, Ito S, Suzuki Y, et al Human serum albumin as an antioxidant in the oxidation of (-)-epigallocatechin gallate: participation of reversible covalent binding for interaction and stabilization. Biosci Biotechnol Biochem. 2011;75(1):100–106. 10.1271/bbb.100600 21228463

[20] Ishii T, Minoda K, Bae MJ, Mori T, Uekusa Y, Ichikawa T, et al Binding affinity of tea catechins for HSA: characterization by high-performance affinity chromatography with immobilized albumin column. Mol Nutr Food Res. 2010;54(6):816–822. 10.1002/mnfr.200900071 20013883

[21] Zinellu A, Sotgia S, Scanu B, Pisanu E, Giordo R, Cossu A, et al Evaluation of non-covalent interactions between serum albumin and green tea catechins by affinity capillary electrophoresis. J Chromatogr A. 2014;1367:167–171. 10.1016/j.chroma.2014.09.053 25294295

[22] Tarcsay Á, Keserű GM. Is there a link between selectivity and binding thermodynamics profiles? Drug Discov. Today. 2015;20(1):86–94. 10.1016/j.drudis.2014.09.014 25263698

[23] Kawasaki Y, Sekiguchi M, Kawasaki M, Hirakura Y. Thermodynamic evaluation of the binding of bisphosphonates to human farnesyl pyrophosphate synthase. Chem Pharm Bull (Tokyo). 2014;62(1):77–83. 10.1248/cpb.c13-00710 24172032

[24] Landrum G. RDKit: Open-source cheminformatics. 2006. Google Scholar. 2006.

[25] Lemmon G, Meiler J. Rosetta Ligand docking with flexible XML protocols. Methods Mol Biol. 2012;819:143–155. 10.1007/978-1-61779-465-0_10 22183535 PMC3749076

[26] Leaver-Fay A, Tyka M, Lewis SM, Lange OF, Thompson J, Jacak R, et al ROSETTA3: an object-oriented software suite for the simulation and design of macromolecules. Methods Enzymol. 2011;487:545–574. 10.1016/B978-0-12-381270-4.00019-6 21187238 PMC4083816

[27] Kuroda D, Gray JJ. Shape complementarity and hydrogen bond preferences in protein-protein interfaces: implications for antibody modeling and protein-protein docking. Bioinformatics. 2016;32(16):2451–2456. 10.1093/bioinformatics/btw197 27153634 PMC4978935

[28] Hubbard SJ, Thornton JM. NACCESS-Computer Program. 1993 Department of Biochemistry and Molecular Biology, University College London.

[29] Zaidi N, Ajmal MR, Rabbani G, Ahmad E, Khan RH. A comprehensive insight into binding of hippuric acid to human serum albumin: a study to uncover its impaired elimination through hemodialysis. PLoS One. 2013;8(8):e71422 10.1371/journal.pone.0071422 23951159 PMC3739763

[30] Martin S, Lamb HK, Brady C, Lefkove B, Bonner MY, Thompson P, et al Inducing apoptosis of cancer cells using small-molecule plant compounds that bind to GRP78. Br J Cancer. 2013;109(2):433–443. 10.1038/bjc.2013.325 23807168 PMC3721410

[31] Wang S, Sun Z, Dong S, Liu Y. Molecular interactions between (-)-epigallocatechin gallate analogs and pancreatic lipase. PLoS One. 2014;9(11):e111143 10.1371/journal.pone.0111143 25365042 PMC4218840

[32] Banerjee T, Singh SK, Kishore N. Binding of naproxen and amitriptyline to bovine serum albumin: biophysical aspects. J Phys Chem B. 2006;110(47):24147–24156. 10.1021/jp062734p 17125386

[33] Murphy KP. Predicting binding energetics from structure: looking beyond DeltaG degrees. Med Res Rev. 1999;19(4):333–339. 10398929 10.1002/(sici)1098-1128(199907)19:4<333::aid-med6>3.0.co;2-5

[34] Spolar RS, Record MT Jr. Coupling of local folding to site-specific binding of proteins to DNA. Science. 1994;263(5148):777–784. 8303294 10.1126/science.8303294

[35] Li M, Hagerman AE. Role of the flavan-3-ol and galloyl moieties in the interaction of (-)-epigallocatechin gallate with serum albumin. J Agric Food Chem. 2014;62(17):3768–3775. 10.1021/jf500246m 24712545 PMC4010290

[36] Pal S, Saha C, Hossain M, Dey SK, Kumar GS. Influence of galloyl moiety in interaction of epicatechin with bovine serum albumin: a spectroscopic and thermodynamic characterization. PLoS One. 2012;7(8):e43321 10.1371/journal.pone.0043321 22916242 PMC3423357

[37] Petitpas I, Bhattacharya AA, Twine S, East M, Curry S. Crystal structure analysis of warfarin binding to human serum albumin: anatomy of drug site I. J Biol Chem. 2001;276(25):22804–22809. 10.1074/jbc.M100575200 11285262

[38] Kyte J, Doolittle RF. A simple method for displaying the hydropathic character of a protein. Journal of Molecular Biology. 1982;157(1):105–132. 10.1016/0022-2836(82)90515-0 7108955

[39] Narukawa M, Noga C, Ueno Y, Sato T, Misaka T, Watanabe T. Evaluation of the bitterness of green tea catechins by a cell-based assay with the human bitter taste receptor hTAS2R39. Biochem Biophys Res Commun. 2011;405(4):620–625. 10.1016/j.bbrc.2011.01.079 21272567

[40] Soares S, Kohl S, Thalmann S, Mateus N, Meyerhof W, De Freitas V. Different phenolic compounds activate distinct human bitter taste receptors. J Agric Food Chem. 2013;61(7):1525–1533. 10.1021/jf304198k 23311874

[41] Sulman FG. TREATMENT OF TRAVELLER'S DIARRHŒA WITH ALBUMIN TANNATE. The Lancet. 1962;280(7256):616 10.1016/S0140-6736(62)90490-7

[42] Ashok PK, Upadhyaya K. Tannins are astringent. J Pharm Phytoc 2012;1(3):45–50.

[43] Xiaokaiti Y, Wu H, Chen Y, Yang H, Duan J, Li X, et al EGCG reverses human neutrophil elastase-induced migration in A549 cells by directly binding to HNE and by regulating alpha1-AT. Sci Rep. 2015;5:11494 10.1038/srep11494 26177797 PMC4503950

[44] Ohtaka H, Velazquez-Campoy A, Xie D, Freire E. Overcoming drug resistance in HIV-1 chemotherapy: the binding thermodynamics of Amprenavir and TMC-126 to wild-type and drug-resistant mutants of the HIV-1 protease. Protein Sci. 2002;11(8):1908–1916. 10.1110/ps.0206402 12142445 PMC2373686

[45] Karas D, Ulrichova J, Valentova K. Galloylation of polyphenols alters their biological activity. Food Chem Toxicol. 2017;105:223–240. 10.1016/j.fct.2017.04.021 28428085

[46] Pettersen EF, Goddard TD, Huang CC, Couch GS, Greenblatt DM, Meng EC, et al UCSF Chimera—a visualization system for exploratory research and analysis. J Comput Chem. 2004;25(13):1605–1612. 10.1002/jcc.20084 15264254

