# Supplementary figures and images for: Thermodynamic and computational analyses reveal the functional roles of the galloyl group of tea catechins in molecular recognition
Source: PLoS One. 2018 Oct 11;13(10):e0204856. doi: 10.1371/journal.pone.0204856 (PMC6181319; doi:10.1371/journal.pone.0204856)

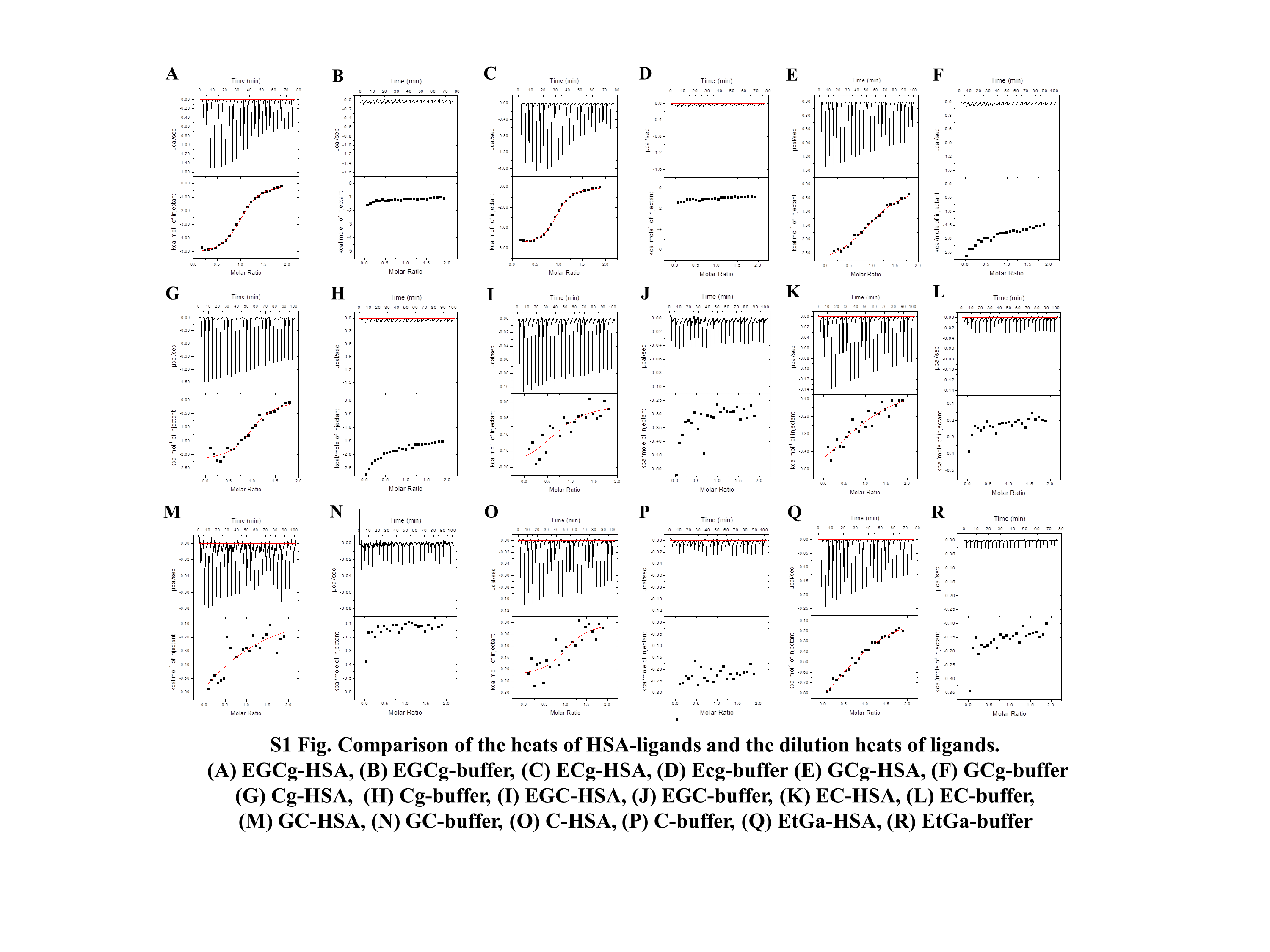

Supplement: S1 Fig — (A) EGCg-HSA, (B) EGCg-buffer, (C) ECg-HSA, (D) Ecg-buffer (E) GCg-HSA, (F) GCg-buffer (G) Cg-HSA, (H) Cg-buffer, (I) EGC-HSA, (J) EGC-buffer, (K) EC-HSA, (L) EC-buffer, (M) GC-HSA, (N) GC-buffer, (O) C-HSA, (P) C-buffer, (Q) EtGa-HSA, (R) EtGa-buffer. (TIF) [file pone.0204856.s001.tif]

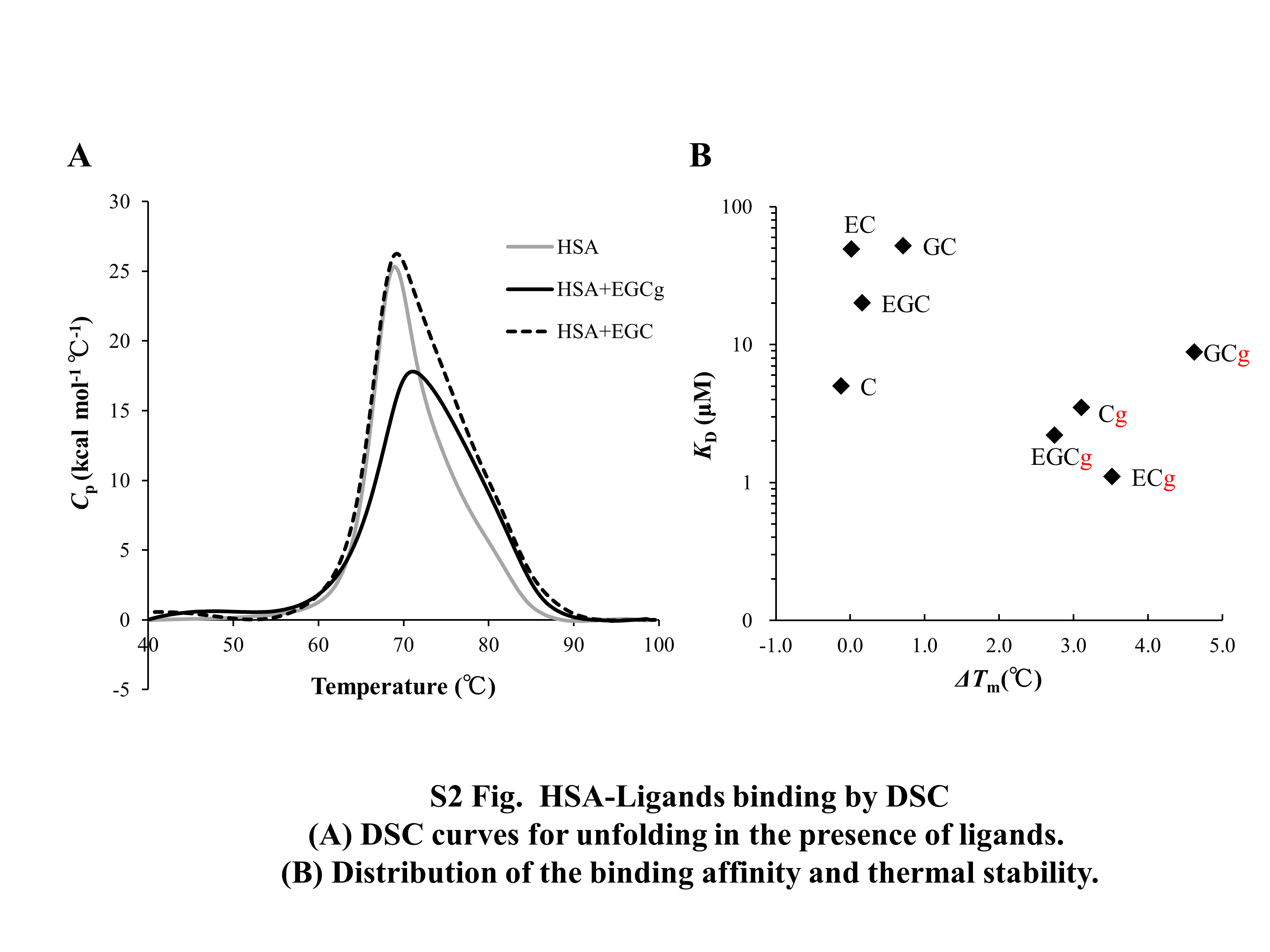

Supplement: S2 Fig — (A) DSC curves for HSA unfolding in the presence of ligands. (B) Distribution of the binding affinity and thermal stability. (TIF) [file pone.0204856.s002.TIF]

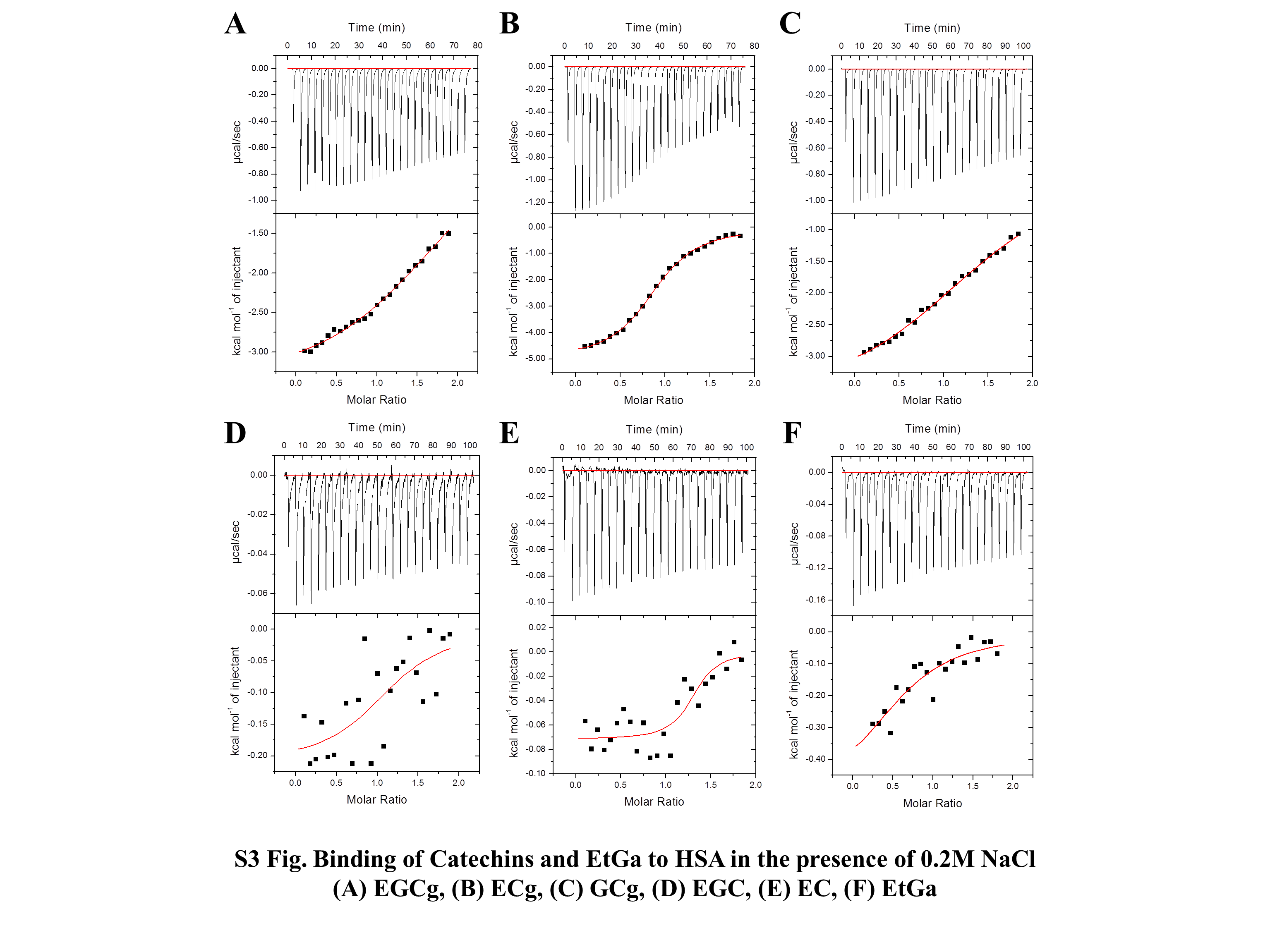

Supplement: S3 Fig — (A) EGCg, (B) ECg, (C) GCg, (D) EGC, (E) EC, (F) EtGa (TIF) [file pone.0204856.s003.TIF]

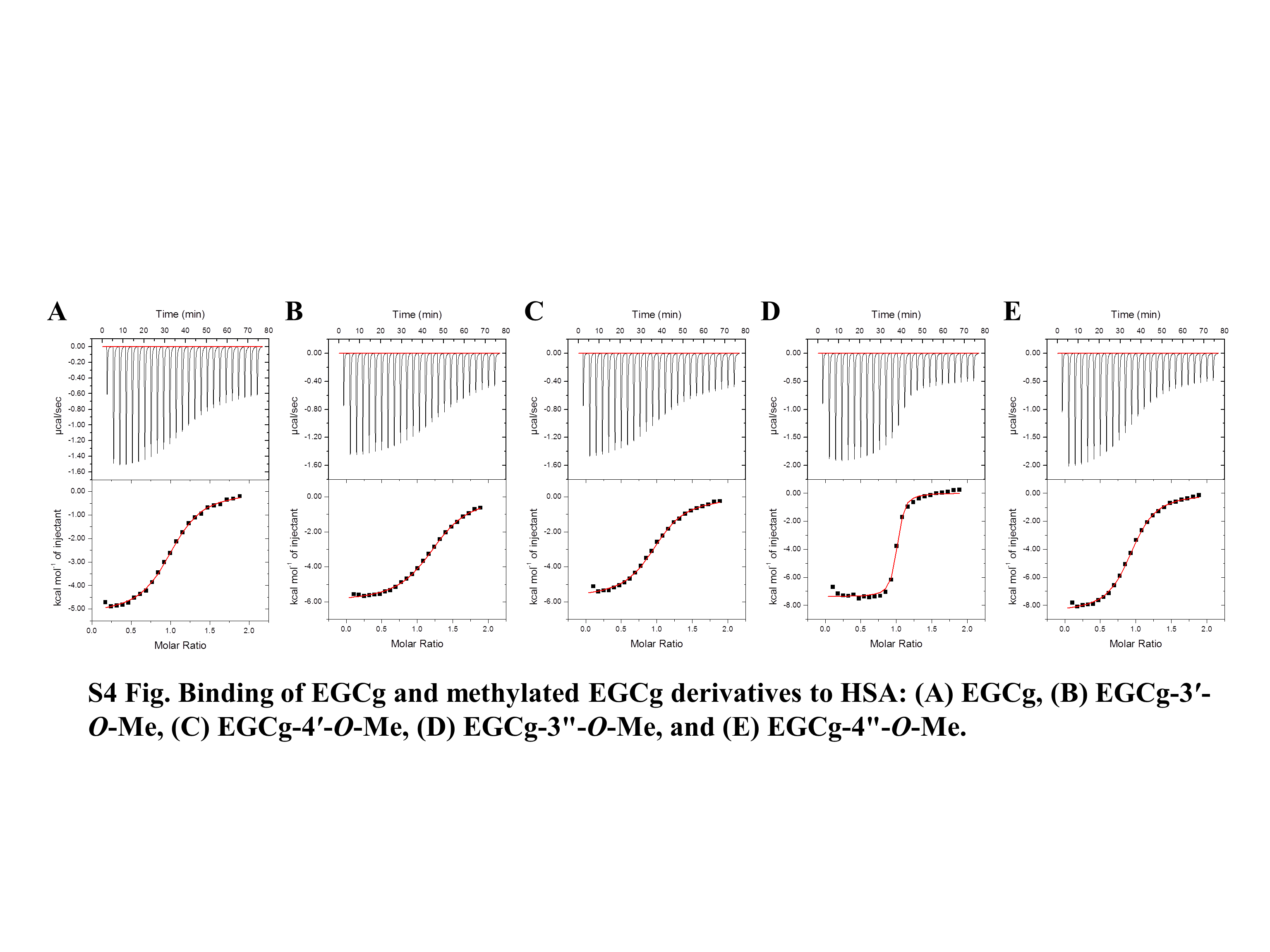

Supplement: S4 Fig — Binding of EGCg and methylated EGCg derivatives to HSA: (A) EGCg, (B)EGCg-3-O-Me, (C) EGCg-4′-O-Me, (D) EGCg-3"-O-Me, and (E) EGCg-4"-O-Me [file pone.0204856.s004.tif]

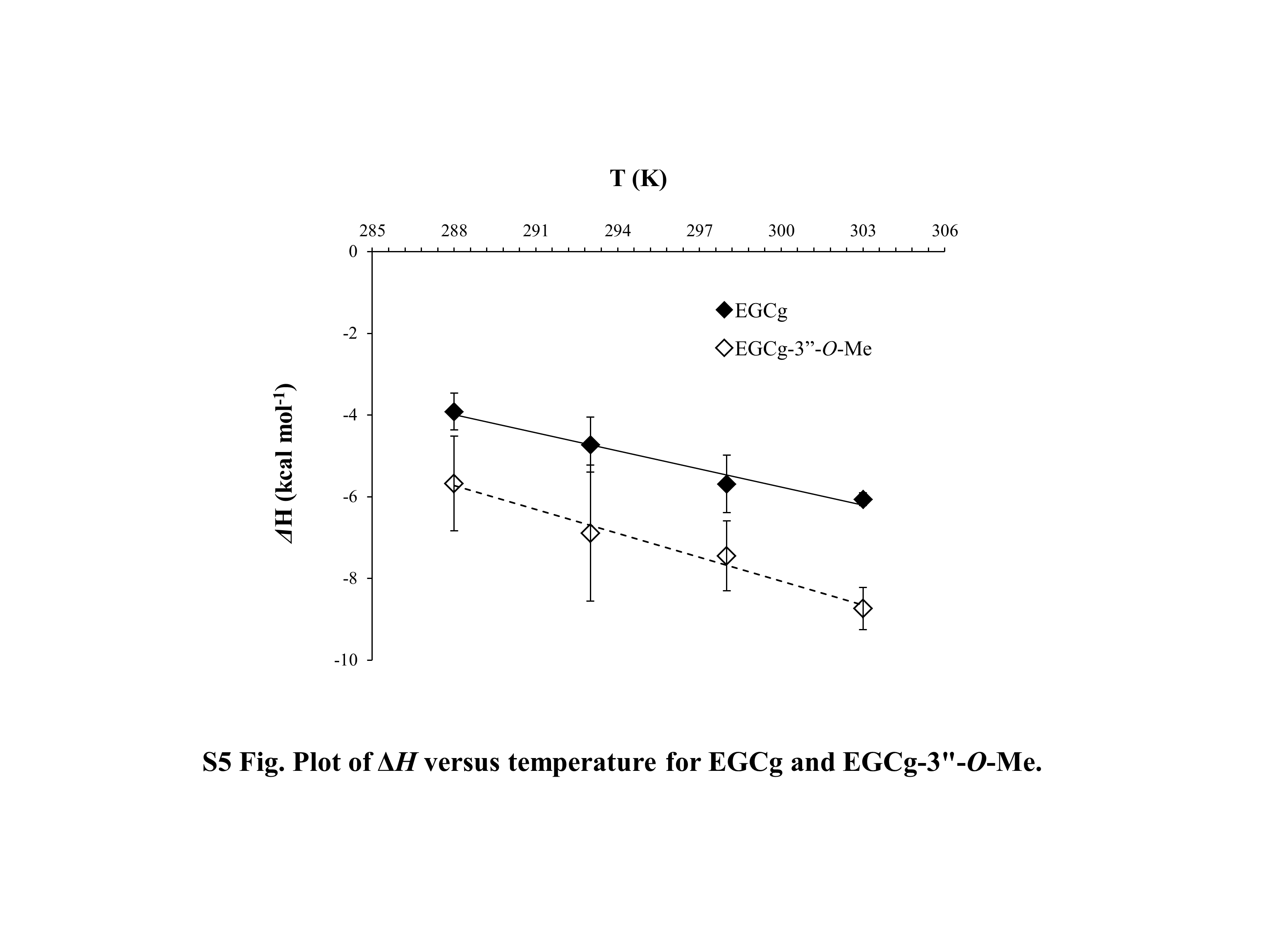

Supplement: S5 Fig — (TIF) [file pone.0204856.s005.TIF]

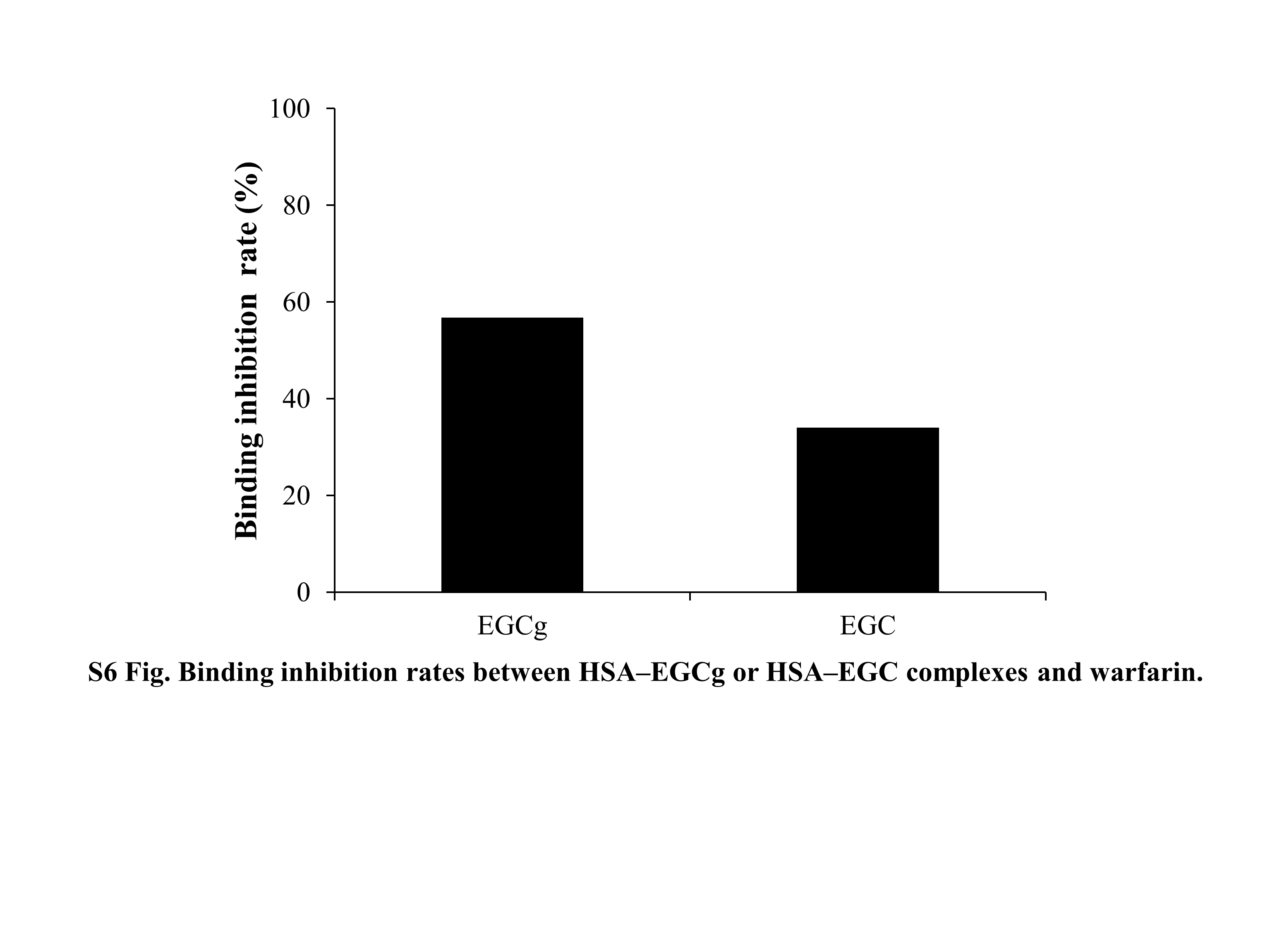

Supplement: S6 Fig — (TIF) [file pone.0204856.s006.TIF]

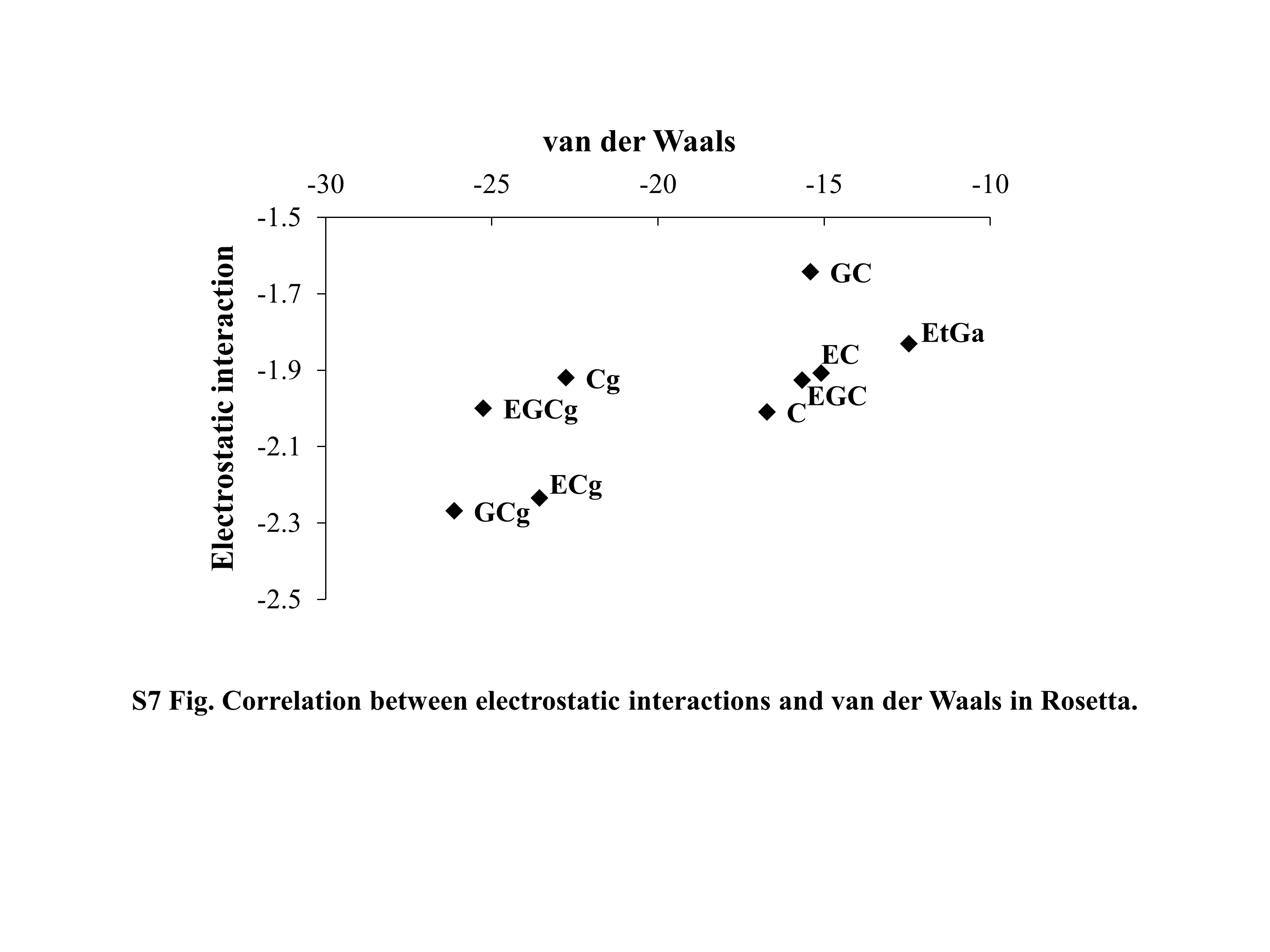

Supplement: S7 Fig — (TIF) [file pone.0204856.s007.tif]

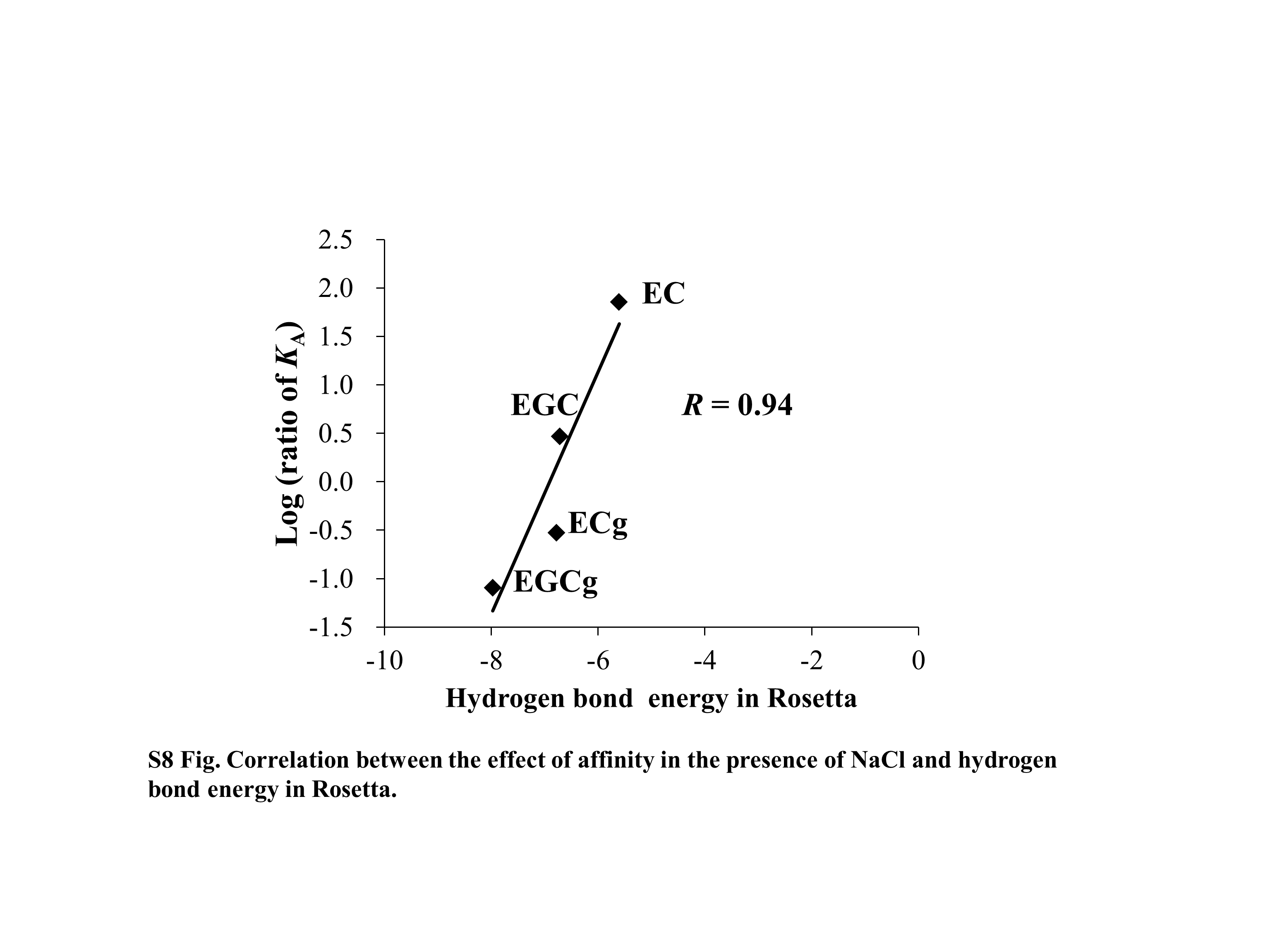

Supplement: S8 Fig — (TIF) [file pone.0204856.s008.tif]

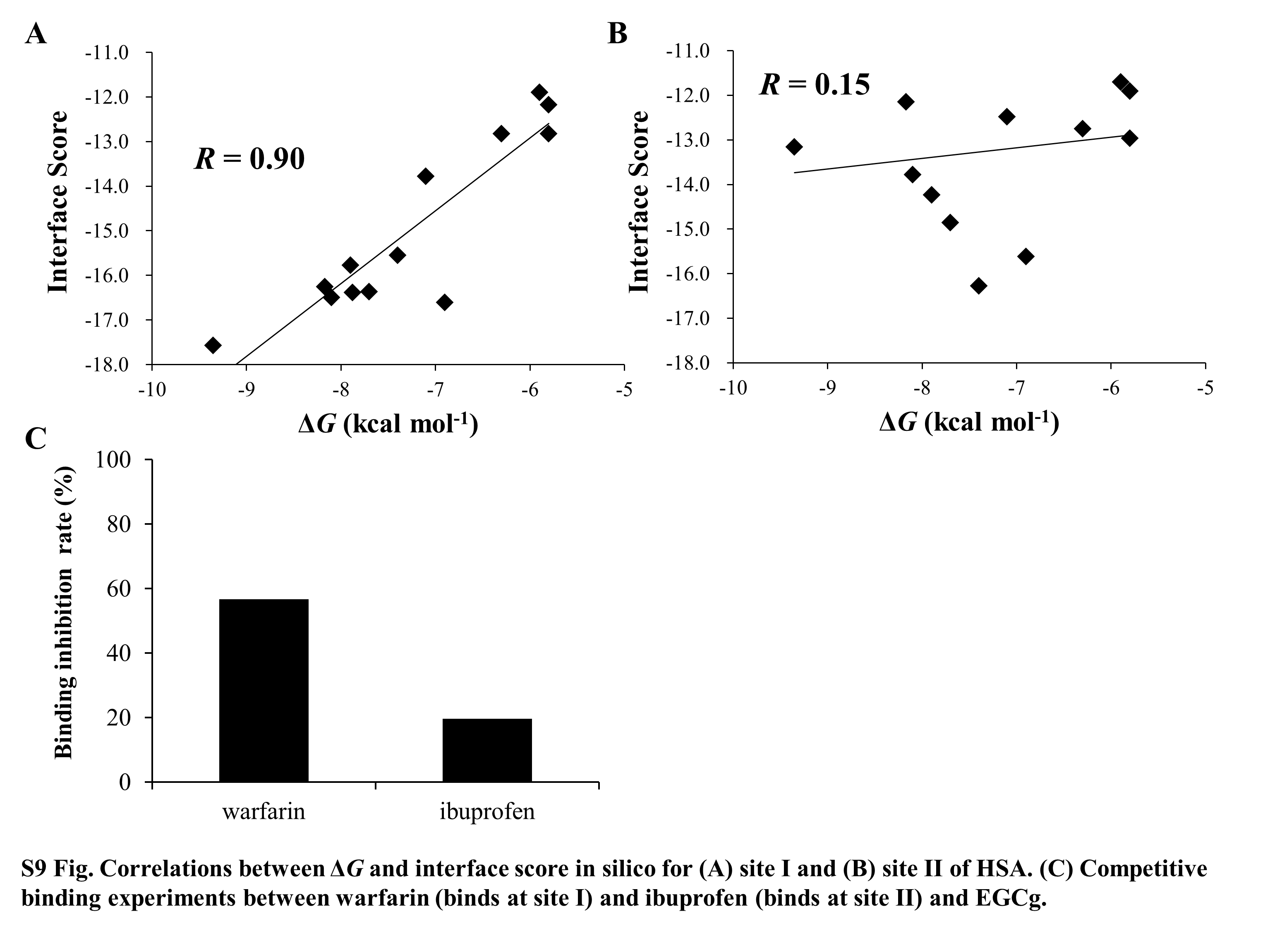

Supplement: S9 Fig — Correlations between ΔG and interface score in silico for (A) site I and (B) site II of HSA. (C) Competitive binding experiments between warfarin (binds at site I) and ibuprofen (binds at site II) and EGCg [file pone.0204856.s009.TIF]

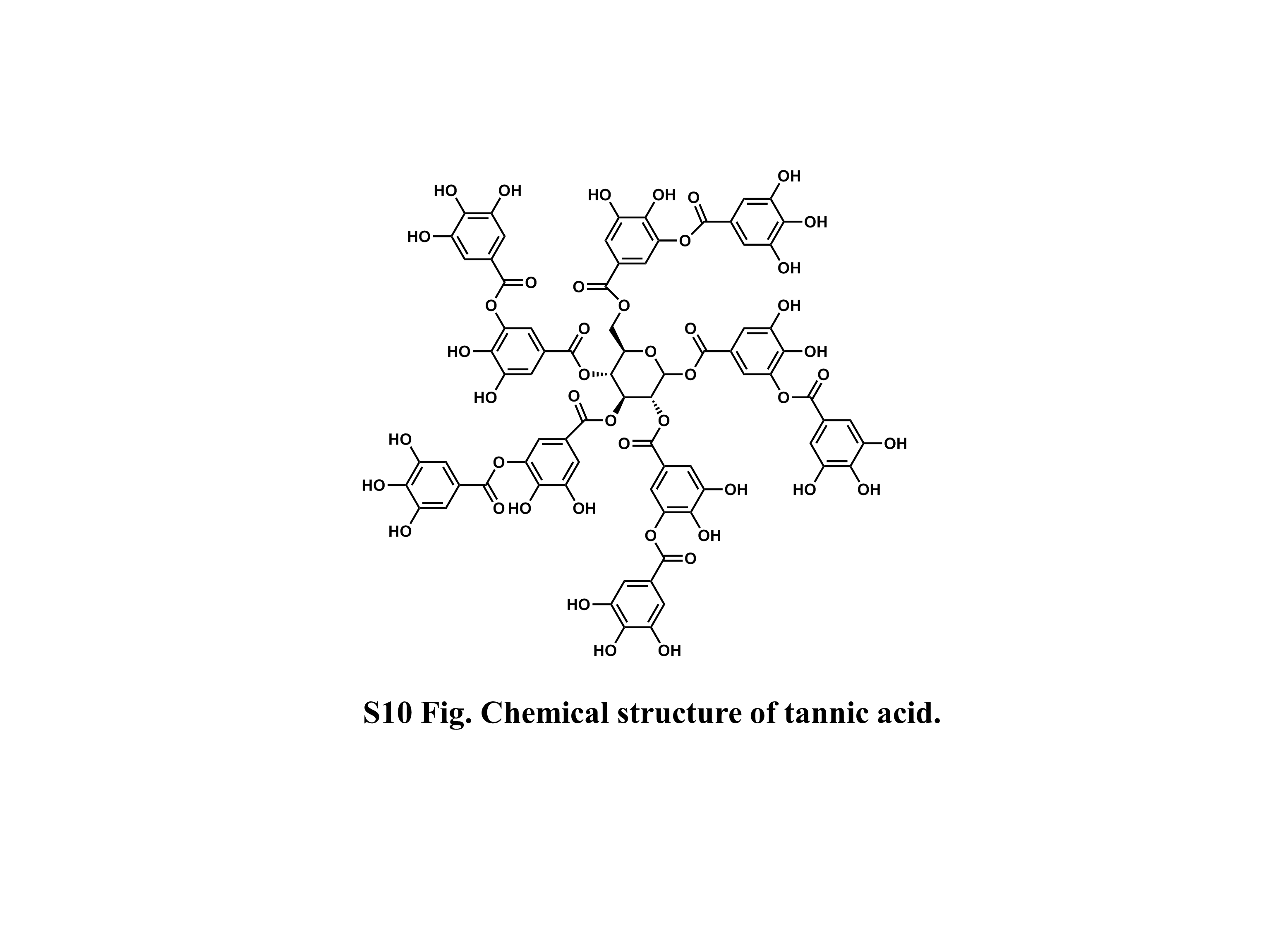

Supplement: S10 Fig — (TIF) [file pone.0204856.s010.tif]
