# Supplementary material for: Thermodynamic and computational analyses reveal the functional roles of the galloyl group of tea catechins in molecular recognition
Source: PLoS One. 2018 Oct 11;13(10):e0204856. doi: 10.1371/journal.pone.0204856 (PMC6181319; doi:10.1371/journal.pone.0204856)
Supplement: S1 Table — (PDF) [file pone.0204856.s011.pdf]

S1 Table. Thermal stabilities and binding affinities of HSA and HSA–catechin complexes [a]

Compound	T_m (°C)	ΔT_m (°C)	K_D (μM)
HSA	68.6 ± 0.1	—	—
HSA–EGCg	71.4 ± 0.3	2.7	2.2 ± 0.3
HSA–ECg	72.2 ± 0.1	3.5	1.1 ± 0.1
HSA–GCg	73.3 ± 0.2	4.6	8.8 ± 3.4
HSA–Cg	71.7 ± 0.1	3.1	3.5 ± 0.5
HSA–EGC	68.8± 0.2	0.2	20 ± 16
HSA–EC	68.7 ± 0.3	0.0	49 ± 18[b]
HSA–GC	69.3 ± 0.3	0.7	52 ± 16[b]
HSA–C	68.5 ± 0.1	−0.1	5.0 ± 8.6

[a] Each value is the average of at least three independent measurements.

[b] The binding stoichiometry was fixed to $N = 1$.
