# Supplementary material for: Thermodynamic and computational analyses reveal the functional roles of the galloyl group of tea catechins in molecular recognition
Source: PLoS One. 2018 Oct 11;13(10):e0204856. doi: 10.1371/journal.pone.0204856 (PMC6181319; doi:10.1371/journal.pone.0204856)
Supplement: S2 Table — (PDF) [file pone.0204856.s012.pdf]

S2 Table. Binding affinities of catechins and EtGa for HSA in the presence and absence of NaCl

Compound	NaCl (M)	K_D (μM)
EGCg	0	2.2
	0.2	27.3
ECg	0	1.1
	0.2	3.7
GCg	0	8.8
	0.2	18.6
EGC	0	19.5
	0.2	6.7
EC	0	48.7
	0.2	0.7
EtGa	0	45.7
	0.2	16.6
