# Supplementary material for: Thermodynamic and computational analyses reveal the functional roles of the galloyl group of tea catechins in molecular recognition
Source: PLoS One. 2018 Oct 11;13(10):e0204856. doi: 10.1371/journal.pone.0204856 (PMC6181319; doi:10.1371/journal.pone.0204856)
Supplement: S3 Table — (PDF) [file pone.0204856.s013.pdf]

S3 Table. Temperature dependence of binding parameters for the interaction of EGCg and EGCg-3″-*O*-Me with HSA

Compound	Temperature (K)	N	K_D (μM)	ΔH (kcal mol^{-1})	$-T\Delta S$ (kcal mol^{-1})	ΔG (kcal mol^{-1})	ΔC_p (cal mol^{-1} K^{-1})
EGCg	288	1.1±0.1	2.3±0.1	-3.9±0.5	-3.5±0.5	-7.4±0.1	−148 [a]
	293	1.1±0.1	2.1±0.7	-4.7±0.7	-2.9±0.8	-7.6±0.7	
	298	1.1±0.1	2.0±0.4	-5.7±0.7	-2.1±0.6	-7.8±0.4	
	303	1.1±0.1	2.1±0.8	-6.1±0.2	-1.8±0.3	-7.9±0.8	
EGCg-3″-*O*-Me	288	1.1±0.1	0.19±0.04	-5.7±1.2	-3.2±1.2	-8.9±0.1	−195 [a]
	293	1.0±0.1	0.12±0.02	-6.9±1.7	-2.4±1.7	-9.3±0.1	
	298	1.0±0.1	0.14±0.02	-7.4±0.9	-1.9±0.9	-9.4±0.1	
	303	1.1±0.1	0.16±0.04	-8.7±0.5	-0.7±0.4	-9.4±0.2	

[a] The ΔC_p value was obtained between 288 and 303
