# Supplementary material for: Thermodynamic and computational analyses reveal the functional roles of the galloyl group of tea catechins in molecular recognition
Source: PLoS One. 2018 Oct 11;13(10):e0204856. doi: 10.1371/journal.pone.0204856 (PMC6181319; doi:10.1371/journal.pone.0204856)
Supplement: S4 Table — (PDF) [file pone.0204856.s014.pdf]

S4 Table. Δ*G* and interface scores for the interaction of catechins and EtGa with HSA

Compound	Δ*G* (kcal mol^{-1})	Interface score
EGCg	−7.7	−16.4
ECg	−8.1	−16.5
GCg	−6.9	−16.6
Cg	−7.4	−15.6
EGC	−6.3	−12.8
EC	−5.8	−12.2
GC	−5.8	−12.8
C	−7.1	−13.8
EtGa	−5.9	−11.9
EGCg-3′-*O*-Me	−7.7	−15.8
EGCg-4′-*O*-Me	−7.9	−16.4
EGCg-3″-*O*-Me	−9.4	−17.6
EGCg-4″-*O*-Me	−8.2	−16.3
